# 1,4-Dibromo­naphthalene-2,3-diol

**DOI:** 10.1107/S1600536811026997

**Published:** 2011-07-13

**Authors:** Qinghe Gao, Yanping Zhu

**Affiliations:** a Key Laboratory of Pesticide and Chemical Biology of Ministry of Education, College of Chemistry, Central China Normal University, Wuhan 430079, People’s Republic of China

## Abstract

In the title compound (r.m.s. deviation for the non-H atoms = 0.020 Å), C_10_H_6_Br_2_O_2_, an intra­molecular O—H⋯O hydrogen bond generates an *S*(6) ring. In the crystal, the same H atom also forms an inter­molecular O—H⋯O hydrogen bond, generating a *C*(2) chain propagating in [100]. The other O—H hydrogen forms a weak O—H⋯π inter­action, and short Br⋯Br contacts [3.5972 (9) Å] also occur.

## Related literature

For the synthesis, see: Lai *et al.* (1993 ▶). For a related structure, see: Ahn *et al.* (2009 ▶).
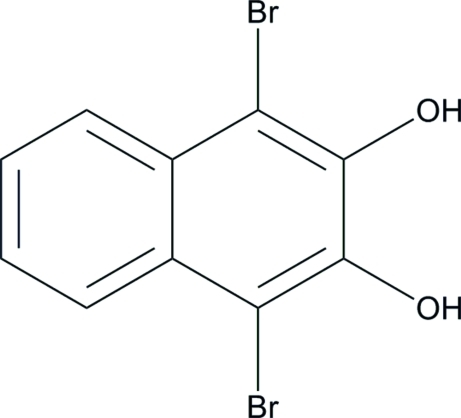


## Experimental

### 

#### Crystal data

C_10_H_6_Br_2_O_2_
*M* *_r_* = 317.96
Orthorhombic, 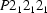

*a* = 5.0928 (9) Å
*b* = 11.932 (2) Å
*c* = 15.779 (3) Å
*V* = 958.9 (3) Å^3^
*Z* = 4
Mo *K*α radiation
μ = 8.42 mm^−1^
*T* = 298 K
0.16 × 0.12 × 0.10 mm

#### Data collection

Bruker SMART APEX CCD diffractometer
Absorption correction: multi-scan (*SADABS*; Bruker, 2001 ▶) *T*_min_ = 0.356, *T*_max_ = 0.486
6339 measured reflections
2363 independent reflections
2156 reflections with *I* > 2σ(*I*)
*R*_int_ = 0.039

#### Refinement

*R*[*F* ^2^ > 2σ(*F* ^2^)] = 0.029
*wR*(*F* ^2^) = 0.068
*S* = 1.00
2363 reflections
133 parameters
2 restraints
H atoms treated by a mixture of independent and constrained refinement
Δρ_max_ = 0.37 e Å^−3^
Δρ_min_ = −0.40 e Å^−3^
Absolute structure: Flack (1983 ▶), 899 Friedel pairs
Flack parameter: 0.034 (15)

### 

Data collection: *SMART* (Bruker, 2001 ▶); cell refinement: *SAINT-Plus* (Bruker, 2001 ▶); data reduction: *SAINT-Plus*; program(s) used to solve structure: *SHELXS97* (Sheldrick, 2008 ▶); program(s) used to refine structure: *SHELXL97* (Sheldrick, 2008 ▶); molecular graphics: *PLATON* (Spek, 2009 ▶); software used to prepare material for publication: *PLATON*.

## Supplementary Material

Crystal structure: contains datablock(s) I, global. DOI: 10.1107/S1600536811026997/hb5940sup1.cif

Structure factors: contains datablock(s) I. DOI: 10.1107/S1600536811026997/hb5940Isup2.hkl

Supplementary material file. DOI: 10.1107/S1600536811026997/hb5940Isup3.cml

Additional supplementary materials:  crystallographic information; 3D view; checkCIF report

## Figures and Tables

**Table 1:** Hydrogen-bond geometry (Å, °) *Cg*1 is the centroid of the C1–C6 ring.

*D*—H⋯*A*	*D*—H	H⋯*A*	*D*⋯*A*	*D*—H⋯*A*
O1—H1*A*⋯*Cg*1^i^	0.82 (1)	2.94 (5)	3.441 (3)	122 (4)
O2—H2*A*⋯O2^ii^	0.81 (1)	2.26 (2)	3.038 (3)	161 (4)
O2—H2*A*⋯O1	0.81 (1)	2.24 (4)	2.653 (4)	112 (4)

Symmetry codes: (i) 
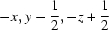
; (ii) 
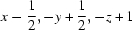
.

**Fig. 1.**
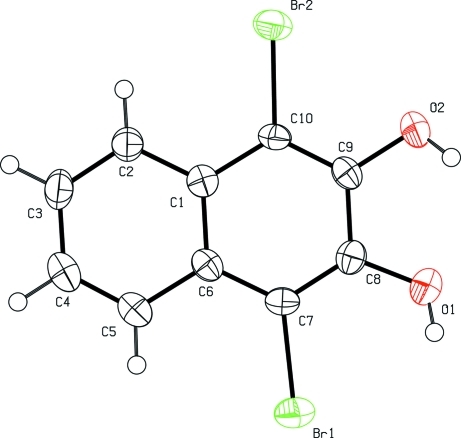
A view of (I), with displacement ellipsoids drawn at the 30% probability level.

## supplementary crystallographic information

### Experimental

The title compound was synthesized according to the literature method (Lai *et al.*, 1993). Crystals of (I) were grown by slow evaporation of a chloroform-methanol (5:1) solution at room temperature.

### Refinement

All H atoms were positioned in geometrically idealized positions and constrained to ride on their parent atoms, with C—H distances in the range 0.93–0.98 Å and *U*_iso_(H) = 1.2*U*_eq_(C) or *U*_iso_(H) =1.5*U*_eq_(C).

### Crystal data

**Table:**

C_10_H_6_Br_2_O_2_	*F*(000) = 608
*M**_r_* = 317.96	*D*_x_ = 2.203 Mg m^−^^3^
Orthorhombic, *P*2_1_2_1_2_1_	Mo *K*α radiation, λ = 0.71073 Å
Hall symbol: P 2ac 2ab	Cell parameters from 3125 reflections
*a* = 5.0928 (9) Å	θ = 2.6–27.3°
*b* = 11.932 (2) Å	µ = 8.42 mm^−^^1^
*c* = 15.779 (3) Å	*T* = 298 K
*V* = 958.9 (3) Å^3^	Block, colorless
*Z* = 4	0.16 × 0.12 × 0.10 mm

### Data collection

**Table:**

Bruker SMART APEX CCD diffractometer	2363 independent reflections
Radiation source: fine-focus sealed tube	2156 reflections with *I* > 2σ(*I*)
graphite	*R*_int_ = 0.039
phi and ω scans	θ_max_ = 28.3°, θ_min_ = 2.1°
Absorption correction: multi-scan (*SADABS*; Bruker, 2001)	*h* = −6→5
*T*_min_ = 0.356, *T*_max_ = 0.486	*k* = −15→15
6339 measured reflections	*l* = −18→21

### Refinement

**Table:**

Refinement on *F*^2^	Secondary atom site location: difference Fourier map
Least-squares matrix: full	Hydrogen site location: inferred from neighbouring sites
*R*[*F*^2^ > 2σ(*F*^2^)] = 0.029	H atoms treated by a mixture of independent and constrained refinement
*wR*(*F*^2^) = 0.068	*w* = 1/[σ^2^(*F*_o_^2^) + (0.0277*P*)^2^] where *P* = (*F*_o_^2^ + 2*F*_c_^2^)/3
*S* = 1.00	(Δ/σ)_max_ = 0.001
2363 reflections	Δρ_max_ = 0.37 e Å^−^^3^
133 parameters	Δρ_min_ = −0.40 e Å^−^^3^
2 restraints	Absolute structure: Flack (1983), 899 Friedel pairs
Primary atom site location: structure-invariant direct methods	Flack parameter: 0.034 (15)

### Special details

**Table:**

Geometry. All e.s.d.'s (except the e.s.d. in the dihedral angle between two l.s. planes) are estimated using the full covariance matrix. The cell e.s.d.'s are taken into account individually in the estimation of e.s.d.'s in distances, angles and torsion angles; correlations between e.s.d.'s in cell parameters are only used when they are defined by crystal symmetry. An approximate (isotropic) treatment of cell e.s.d.'s is used for estimating e.s.d.'s involving l.s. planes.
Refinement. Refinement of *F*^2^ against ALL reflections. The weighted *R*-factor *wR* and goodness of fit *S* are based on *F*^2^, conventional *R*-factors *R* are based on *F*, with *F* set to zero for negative *F*^2^. The threshold expression of *F*^2^ > σ(*F*^2^) is used only for calculating *R*-factors(gt) *etc*. and is not relevant to the choice of reflections for refinement. *R*-factors based on *F*^2^ are statistically about twice as large as those based on *F*, and *R*- factors based on ALL data will be even larger.

### Fractional atomic coordinates and isotropic or equivalent isotropic displacement parameters (Å^2^)

**Table:**

	*x*	*y*	*z*	*U*_iso_*/*U*_eq_	
Br1	−0.18029 (8)	0.17357 (3)	0.16514 (2)	0.04109 (12)	
Br2	0.61683 (7)	0.45789 (3)	0.40915 (2)	0.04028 (12)	
C1	0.4056 (7)	0.3970 (3)	0.24593 (19)	0.0273 (7)	
C2	0.5849 (7)	0.4724 (3)	0.2089 (2)	0.0331 (7)	
H2	0.7024	0.5113	0.2431	0.040*	
C3	0.5879 (8)	0.4891 (3)	0.1222 (2)	0.0409 (9)	
H3	0.7077	0.5386	0.0982	0.049*	
C4	0.4101 (8)	0.4313 (3)	0.0709 (2)	0.0423 (9)	
H4	0.4103	0.4444	0.0128	0.051*	
C5	0.2376 (8)	0.3569 (3)	0.1037 (2)	0.0360 (8)	
H5	0.1239	0.3183	0.0679	0.043*	
C6	0.2291 (7)	0.3372 (3)	0.19325 (18)	0.0285 (7)	
C7	0.0531 (7)	0.2609 (3)	0.23054 (18)	0.0289 (7)	
C8	0.0467 (7)	0.2438 (3)	0.31682 (19)	0.0291 (7)	
C9	0.2215 (7)	0.3043 (3)	0.37007 (19)	0.0285 (7)	
C10	0.3945 (7)	0.3778 (3)	0.33519 (19)	0.0267 (6)	
O1	−0.1160 (6)	0.1729 (2)	0.35854 (16)	0.0424 (6)	
H1A	−0.251 (5)	0.151 (4)	0.336 (3)	0.064*	
O2	0.2141 (6)	0.2872 (2)	0.45569 (15)	0.0389 (6)	
H2A	0.072 (5)	0.261 (4)	0.468 (3)	0.058*	

### Atomic displacement parameters (Å^2^)

**Table:**

	*U*^11^	*U*^22^	*U*^33^	*U*^12^	*U*^13^	*U*^23^
Br1	0.0417 (2)	0.0386 (2)	0.04299 (19)	−0.00633 (18)	−0.00583 (16)	−0.00902 (16)
Br2	0.0427 (2)	0.0432 (2)	0.03491 (16)	−0.00489 (18)	−0.00972 (15)	−0.00380 (15)
C1	0.0291 (18)	0.0237 (15)	0.0290 (14)	0.0050 (14)	0.0019 (14)	−0.0014 (12)
C2	0.0373 (19)	0.0308 (18)	0.0311 (14)	−0.0022 (16)	0.0035 (14)	−0.0004 (14)
C3	0.046 (2)	0.041 (2)	0.0359 (16)	−0.0081 (18)	0.0107 (16)	0.0062 (16)
C4	0.051 (2)	0.048 (2)	0.0274 (15)	0.002 (2)	0.0016 (15)	0.0048 (15)
C5	0.042 (2)	0.0394 (19)	0.0269 (15)	0.0025 (16)	−0.0019 (14)	−0.0007 (14)
C6	0.0333 (19)	0.0274 (16)	0.0248 (13)	0.0058 (15)	0.0004 (12)	−0.0010 (13)
C7	0.0299 (18)	0.0271 (17)	0.0296 (15)	−0.0002 (14)	−0.0038 (13)	−0.0058 (13)
C8	0.0304 (18)	0.0238 (16)	0.0332 (15)	0.0018 (14)	0.0028 (13)	0.0027 (13)
C9	0.0329 (19)	0.0290 (17)	0.0235 (13)	0.0054 (14)	−0.0009 (13)	0.0020 (12)
C10	0.0298 (16)	0.0245 (15)	0.0256 (13)	−0.0004 (14)	−0.0059 (14)	−0.0037 (12)
O1	0.0440 (16)	0.0422 (15)	0.0408 (13)	−0.0125 (14)	0.0013 (12)	0.0070 (11)
O2	0.0422 (16)	0.0486 (15)	0.0258 (10)	−0.0055 (13)	0.0010 (10)	0.0066 (11)

### Geometric parameters (Å, °)

**Table:**

Br1—C7	1.888 (3)	C5—C6	1.434 (4)
Br2—C10	1.886 (3)	C5—H5	0.9300
C1—C2	1.409 (5)	C6—C7	1.407 (5)
C1—C6	1.417 (5)	C7—C8	1.377 (4)
C1—C10	1.428 (4)	C8—O1	1.355 (4)
C2—C3	1.383 (4)	C8—C9	1.421 (5)
C2—H2	0.9300	C9—C10	1.359 (5)
C3—C4	1.397 (5)	C9—O2	1.367 (4)
C3—H3	0.9300	O1—H1A	0.819 (10)
C4—C5	1.352 (5)	O2—H2A	0.810 (10)
C4—H4	0.9300		
			
C2—C1—C6	119.3 (3)	C7—C6—C5	122.5 (3)
C2—C1—C10	122.5 (3)	C1—C6—C5	118.5 (3)
C6—C1—C10	118.2 (3)	C8—C7—C6	121.6 (3)
C3—C2—C1	120.6 (3)	C8—C7—Br1	116.4 (3)
C3—C2—H2	119.7	C6—C7—Br1	122.0 (2)
C1—C2—H2	119.7	O1—C8—C7	126.0 (3)
C2—C3—C4	119.7 (3)	O1—C8—C9	114.4 (3)
C2—C3—H3	120.1	C7—C8—C9	119.6 (3)
C4—C3—H3	120.1	C10—C9—O2	121.0 (3)
C5—C4—C3	121.6 (3)	C10—C9—C8	119.6 (3)
C5—C4—H4	119.2	O2—C9—C8	119.4 (3)
C3—C4—H4	119.2	C9—C10—C1	121.9 (3)
C4—C5—C6	120.3 (3)	C9—C10—Br2	117.7 (2)
C4—C5—H5	119.9	C1—C10—Br2	120.4 (2)
C6—C5—H5	119.9	C8—O1—H1A	120 (3)
C7—C6—C1	119.0 (3)	C9—O2—H2A	108 (3)
			
C6—C1—C2—C3	0.7 (5)	Br1—C7—C8—O1	−2.0 (5)
C10—C1—C2—C3	−179.5 (3)	C6—C7—C8—C9	0.0 (5)
C1—C2—C3—C4	0.5 (6)	Br1—C7—C8—C9	178.4 (2)
C2—C3—C4—C5	−1.6 (6)	O1—C8—C9—C10	179.8 (3)
C3—C4—C5—C6	1.4 (6)	C7—C8—C9—C10	−0.5 (5)
C2—C1—C6—C7	179.1 (3)	O1—C8—C9—O2	0.3 (5)
C10—C1—C6—C7	−0.7 (5)	C7—C8—C9—O2	179.9 (3)
C2—C1—C6—C5	−0.8 (5)	O2—C9—C10—C1	180.0 (3)
C10—C1—C6—C5	179.4 (3)	C8—C9—C10—C1	0.4 (5)
C4—C5—C6—C7	179.8 (3)	O2—C9—C10—Br2	−1.7 (4)
C4—C5—C6—C1	−0.2 (5)	C8—C9—C10—Br2	178.8 (2)
C1—C6—C7—C8	0.6 (5)	C2—C1—C10—C9	−179.6 (3)
C5—C6—C7—C8	−179.5 (3)	C6—C1—C10—C9	0.2 (5)
C1—C6—C7—Br1	−177.7 (2)	C2—C1—C10—Br2	2.0 (4)
C5—C6—C7—Br1	2.2 (5)	C6—C1—C10—Br2	−178.1 (2)
C6—C7—C8—O1	179.6 (3)		

### Hydrogen-bond geometry (Å, °)

**Table:**

Cg1 is the centroid of the C1–C6 ring.

**Table:**

*D*—H···*A*	*D*—H	H···*A*	*D*···*A*	*D*—H···*A*
O1—H1A···Cg1^i^	0.82 (1)	2.94 (5)	3.441 (3)	122 (4)
O2—H2A···O2^ii^	0.81 (1)	2.26 (2)	3.038 (3)	161 (4)
O2—H2A···O1	0.81 (1)	2.24 (4)	2.653 (4)	112 (4)

Symmetry codes: (i) −*x*, *y*−1/2, −*z*+1/2; (ii) *x*−1/2, −*y*+1/2, −*z*+1.
